# Nonsurgical Management of Severe Osteonecrosis of the Knee in an HIV-Positive Patient: A Case Report

**DOI:** 10.1155/2011/935041

**Published:** 2011-07-02

**Authors:** Stephanie A. Nixon, Kelly K. O'Brien, Gary Rubin

**Affiliations:** ^1^Department of Physical Therapy, University of Toronto, 160-500 University Avenue, Toronto, ON, Canada M5G 1V7; ^2^International Centre for Disability and Rehabilitation (ICDR), 160-500 University Avenue, Toronto, ON, Canada M5G 1V7; ^3^Health Economics and HIV/AIDS Research Division (HEARD), University of KwaZulu-Natal, Durban 4041, South Africa; ^4^School of Rehabilitation Science, McMaster University, 1400 Main Street West, Room 403, Hamilton, ON, Canada L8S 1C7; ^5^Department of Family and Community Medicine, University of Toronto, Toronto, ON, Canada M5T 1W7; ^6^Department of Medicine, HIV Clinical Research Unit, Sunnybrook Health Sciences Center, 406-235 Danforth Avenue, Toronto, ON, Canada M4K 1N2

## Abstract

Due to the life-prolonging effects of combination antiretroviral therapy, many people with HIV are living longer. However, this enhanced longevity is often mirrored by increased disability resulting from HIV and/or the adverse effects of medication. Management of HIV-positive patients is further complicated by comorbidities related to aging, including bone and joint disorders. In this paper, we describe the nonsurgical management of an HIV-positive patient with premature onset of severe osteonecrosis of the knee. A 50-year-old man who had been HIV-positive for 16 years and on combination antiretroviral therapy for 11 years presented to his family physician with extreme discomfort in his right knee. He was diagnosed with osteonecrosis of the right knee, but resisted total knee arthroplasty because of potential complications under anesthesia related to comorbid advanced liver disease. Instead, a successful combination of non-surgical management strategies was employed by the patient and his health care team.

## 1. Introduction

Due to the life prolonging effects of combination antiretroviral therapy, people with HIV are living longer [1, 2]. This increased longevity has been mirrored by a rise in disability resulting from primary HIV infection and the potentially adverse effects of medication [3–5]. Management of HIV-positive patients is further complicated by concurrent health conditions (or comorbidities) related to aging, which are occurring as these clients live longer lives, including cardiovascular disease, liver disease, kidney disease, diabetes, cancers, and challenges to mental health [4, 6, 7].

In particular, bone and joint disorders such as osteoarthritis, osteopenia, osteoporosis, and osteonecrosis are a growing concern as people live longer with HIV [8]. A systematic review with over 800 participants reported a high prevalence of osteopenia (67%) among HIV-positive participants, of which 15% had osteoporosis, a rate three times greater than HIV-negative controls [9]. Although the underlying mechanism of bone mineral density loss in HIV is unknown, it is likely to be multifactorial, associated with longer duration of HIV infection, increased age, low body weight, higher viral load, smoking, and while somewhat controversial, more recently use of antiretroviral therapy [10–14].

A number of case reports suggest an association between HIV infection and osteonecrosis [15]. Osteonecrosis (or avascular necrosis) results from the ischemic death of bone secondary to a loss of blood supply. Osteonecrosis is typically found in the femoral head which can lead to subchondral collapse and severe osteoarthritis [12, 16]. Incidence of osteonecrosis among people living with HIV is increasing, associated with both the duration of HIV infection and antiretroviral treatment [17].

No known treatment interventions effective in halting bone death associated with osteonecrosis exist [12]. Surgery is one option for HIV-positive patients who are medically stable, including core decompression, bone grafting, osteotomy, and joint arthroplasty [12]. However, HIV-positive patients may wish to avoid surgical intervention for a variety of reasons. First, limited durability of total joint replacements presents a challenge to many HIV-positive patients who are experiencing early onset of joint problems but who expect to live for decades. As such, patients may wish to postpone joint replacement surgery for as long as possible. Second, higher rates of complications such as deep prosthetic infection and aseptic loosening result in a greater need for repeated surgeries among people living with HIV with total joint arthroplasty [18, 19]. Third, comorbidities such as cardiovascular disease, liver disease, and diabetes are increasingly common among HIV-positive patients, which may elevate the risk of complications from surgery, or decrease the priority patients place on their disabling joint conditions.

In this paper, we describe the nonsurgical management of an HIV-positive patient with premature onset of severe osteonecrosis of the knee.

## 2. Case Presentation

### 2.1. Chief Complaint

This case involves a 50-year-old HIV-positive man who presented to his family physician in 2006 with extreme discomfort in his right knee.

### 2.2. Past Medical History

The patient was diagnosed with HIV in 1990 and had been taking combination antiretroviral medication since 1995. Five years prior to the incident of right knee pain, he was diagnosed with osteonecrosis of the left lateral tibia. His rheumatologist prescribed Celebrex which managed the left knee pain well. However, the following year, the patient was diagnosed with cirrhosis of the liver resulting from his antiretroviral medications. Celebrex was discontinued because of the contraindication with liver disease, and Tylenol was initiated for his left knee pain. The patient considered this management plan to be acceptable, particularly in light of his life-threatening liver disease, which became his focus of care. His liver disease had stabilized, and his left knee pain was well managed when the current incident of right knee pain struck.

### 2.3. Current History

The patient presented with severe pain in his right knee and was unable to weight bear with his leg fully extended. Bone scan in February 2006 revealed osteonecrosis of the right knee, osteopenia in both hips, small fractures bilaterally in the second and third metatarsals, and osteonecrosis of the right medial femoral condyle. The endocrinologist prescribed weekly Fosimax, plus a high dose of calcium and vitamin D and K supplements. The patient preferred not to take Tylenol because of his liver disease, and so his rheumatologist prescribed Tramidol taken at night to relieve morning joint pain.

**Figure 1:**
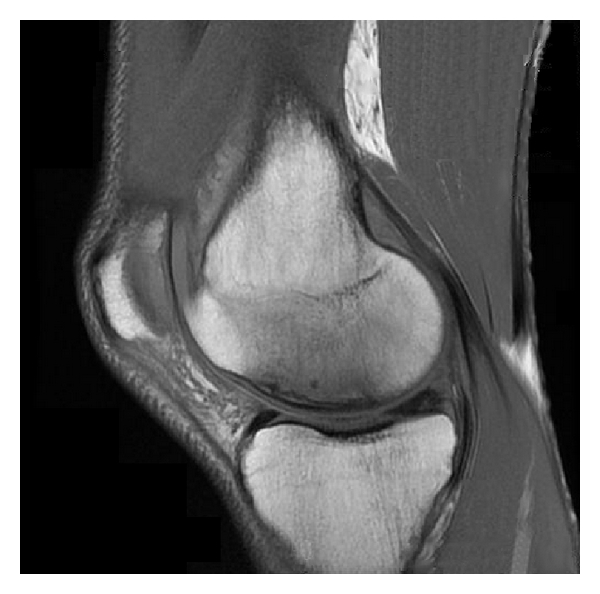
MRI of right knee demonstrating flattening and irregularity of the articular surface with small cyst formation and alteration of the marrow signal, which suggests osteonecrosis.

Nearly one year later, MRI showed osteonecrosis and a medial meniscal tear of the right knee (see Figure 1), and the patient was referred to an orthopedic surgeon to explore surgical options. The surgeon advised that minor debridement was not an option because of the lack of healthy bone, and that a total knee arthroplasty would likely be advised within the next two years. The patient was resistant to the surgical option because of his advanced liver disease and concern for potential complications under anesthesia.

The orthopedic surgeon referred the patient to a physiatrist to fit an unloader brace. The unloader brace, designed to lift the femur off of the tibia for pain relief, was worn for six months, followed by a neoprene brace for six more months. Shortly after fitting the unloader brace, the patient began a one-month course of twice weekly physical therapy for postural retraining and exercises to address the patient's altered knee biomechanics. The physical therapist also delivered manual therapy techniques to resolve the patient's recent onset of left shoulder pain that had resulted from the use of canes for mobility.

Over the next 18 months, the patient engaged in a combination of nonsurgical approaches to manage the pain, limited mobility, and decreased range of motion related to the osteonecrosis of his right knee. First, he began using Nordic walking poles to assist with walking. Nordic walking is an aerobic activity that involves walking with two specially designed poles, similar to cross-country skiing. Evolved from off-season skitraining, Nordic walking simultaneously combines the upper and lower body; the user applies a force through the poles with each stride engaging arm, shoulder, upper chest, back, and core postural muscles. The patient began a daily routine of Nordic walking for 1 kilometer, which took approximately 1 hour. He also initiated a programme of therapeutic yoga 2–3 times per week for 90 minutes for alignment, balance and stability. The yoga instructor, who was also a physical therapist, guided the patient through a combination of stretching and strengthening of his postural muscles. Later that year, the patient began hydrotherapy involving deep-end walking for 30 minutes performed 2-3 times each week. The patient perceived benefits of hydroptherapy during the early phases of his recovery when pain was greatly limiting his abilities to mobilize on land; however, he discontinued this intervention after approximately three months once his function had improved. As a former competitive swimmer, his tendency was to re-engage in more active swimming techniques but he found the whipkick aggravated his knee pain.

At the end of this 18 month period, the patient was able to walk 5 kilometers pain-free without a brace, with the Nordic walking poles within 1 hour. Atrophy in his right quadriceps and gastrocnemius that was present after using the braces one year earlier had resolved. Most importantly, the patient reported greatly reduced knee pain and disability. A repeat MRI taken in 2008 showed that the degeneration of the right knee had stabilized. Discussions of total knee arthroplasty were discontinued.

## 3. Discussion

HIV-positive patients and their health care providers will increasingly be presented with medical management dilemmas as people with HIV live longer lives with the premature onset of bone and joint conditions related to HIV and its medications. For many patients, nonsurgical interventions may be preferred because of comorbidities that elevate the risk of complications. 

Like many others living with HIV, the patient in this case had advanced liver disease. Since the advent of combination antiretroviral therapy, there has been a substantial decrease in deaths related to AIDS. However, liver disease is now the most common non-AIDS-related cause of death among HIV-infected patients, accounting for 14–18% of all deaths in this population and almost half of deaths among hospitalized HIV-infected patients [20]. 

Both aerobic and progressive resistive exercise have been shown to be safe in HIV-positive adults who are medically stable. Aerobic exercise, or a combination of aerobic and resistive exercise, has been linked to improvements in cardiopulmonary fitness (maximum oxygen consumption), body composition (leg muscle area, percent body fat), and psychological status (depression-dejection symptoms) for adults living with HIV [21]. The patient in this case realized positive results from aerobic exercise in the form of Nordic walking, yoga, and hydrotherapy. 

This patient views his discovery of the Nordic walking poles as a turning point in his rehabilitation journey. Nordic walking offers benefits of higher caloric expenditure, oxygen consumption, and heart rate compared to walking without Nordic poles, without an increase in rate of perceived exertion, thus allowing individuals to exercise for a longer duration [22]. Nordic walking has been linked to mobility improvements for people with Parkinson's Disease [23], chronic obstructive pulmonary disease [24], and cardiac rehabilitation [25]. While Nordic walking offered aerobic benefits to the patient in this case, reasons for his simultaneous reduction of knee pain are less clear as Nordic walking has not been found to reduce loading of the knee joint [26]. The use of the unloader brace in this case was also seen to offer an important contribution to pain management and recovery, presumedly by taking pressure off of the joint and allowing for enhanced revascularization. 

The patient also began a programme of yoga. An increasing number of people living with HIV are engaging in complementary and alternative therapy interventions, of which yoga and meditation are popular forms [27]. While evidence in this area is still emerging, yoga has been associated with a reduction in blood pressure among people living with HIV at risk of cardiovascular disease [28]. Case studies have reported reductions in pain and anxiety among people living with HIV who engage in yoga [29]. The effect of yoga on other outcomes, such as body weight, fat mass, lipids, or overall quality of life, are less clear. 

Hydrotherapy was another dimension of this patient's nonsurgical approach to managing his knee pain and decreased function. Water-based therapies hold broad rehabilitative potential, ranging from treatment of acute injuries through to health maintenance in the context of chronic diseases [30]. Although research on the effects of hydrotherapy among people living with HIV is limited [31], its role in relation to bone and joint pathologies is well studied. A 6-week aquatic physical therapy programme with symptomatic hip and knee osteoarthritis (OA) patients demonstrated significantly less pain and improved physical function, strength, and quality of life compared to a control group that received no intervention [32]. When compared to land-based exercises, randomized controlled trials have revealed that water-based exercises were equally effective at improving strength and function, especially among people with osteoarthritis [33–35]. Hydrotherapy has been shown to significantly decrease pain, especially after treatment [34] and activity [33]. These contributions to pain management may be related to the water buoyancy, the reduction of stress on the joints, bones, and muscles, as well as the warmth and hydrostatic pressure, which decreases swelling and improves circulation [30, 34, 36].

Along with these general approaches to wellness, the patient in this case received exercise prescription from a physical therapist who was able to design therapeutic exercises to address his pathologies. While understanding of the role of physical therapy (and other rehabilitation services) in addressing the needs of people living with HIV is increasing, a Canadian survey found that few rehabilitation professionals (defined as occupational therapists, physical therapists and speech-language pathologists) self-identify as caring for people living with HIV [37]. Furthermore, health professionals specializing in HIV in Canada (including physicians and nurses) were found to refer people living with HIV to HIV service organizations and social workers to address participation restrictions and social issues far more often than they referred them to rehabilitation professionals to address impairments and activity limitations [38]. The Canadian Working Group on HIV and Rehabiltiation (www.hivandrehab.ca) is a national working group engaged in interprofessional mentorship, online learning, and other educational activities on HIV and AIDS for rehabilitation professionals. To further address knowledge gaps about HIV and rehabilitation, the Canadian Working Group on HIV and Rehabilitation recently updated a comprehensive resource designed to enhance the capacity of rehabilitation professionals to better serve the needs of people living with HIV [39].

## 4. Conclusion

Bone and joint disorders are becoming increasingly common among HIV-positive patients, particularly as people age with the disease. This case described an HIV-positive patient with severe osteonecrosis of the knee, but for whom a total joint arthroplasty was undesirable because of his advanced liver disease. A combination of nonsurgical management strategies, including Nordic walking, yoga, and hydrotherapy, identified collaboratively by the patient and his health care team, resulted in successful management of his knee pain and related disability while increasing his overall exercise capacity. 

Clinicians should be aware of the unique needs facing HIV-positive clients with bone and joint disorders, and the potential range of nonsurgical interventions that are available to address patients' concerns. For many of these nonsurgical interventions, evidence is still emerging on their effect for people living with HIV. This case report highlights the use of nonsurgical interventions for people living with HIV with premature onset of bone and joint disorders. Future research may use more rigourous research approaches to explore and understand the impact of different nonsurgical interventions on impairments and activity limitations for people living with HIV who have bone and joint disorders.
